# Effects of buccal acupuncture on postoperative analgesia in elderly patients undergoing laparoscopic radical gastrectomy: a randomized controlled trial

**DOI:** 10.3389/fneur.2024.1408360

**Published:** 2024-06-26

**Authors:** Dong-xue Zhu, Yan-ling Yang, Lei Yang, Yuan-yuan Zhao, Ya-yun Xie, Wei Wang, Jie Lv, Wan-you Yu

**Affiliations:** ^1^Department of Anesthesiology, The Affiliated Jiangning Hospital of Nanjing Medical University, Nanjing, China; ^2^Department of Anesthesiology, Wenjiang District People’s Hospital of Chengdu, Chengdu, China; ^3^Department of Anesthesiology, Huainan First People’s Hospital, The First Affiliated Hospital of Anhui University of Science and Technology, Huainan, China

**Keywords:** buccal acupuncture, elderly, laparoscopic radical gastrectomy, analgesia, stress response

## Abstract

**Objective:**

This study aimed to evaluate the efficacy and safety of buccal acupuncture on postoperative analgesia, perioperative stress response and adverse events in elderly patients undergoing laparoscopic radical gastrectomy.

**Methods:**

It was a prospective, outcome assessor-blinded, randomized controlled trial, involving 90 patients aged 65–80 years who were treated with an elective laparoscopic radical gastrectomy. They were randomly assigned to buccal acupuncture group (Group B) and control group (Group C). Buccal acupuncture was applied to patients of Group B before the induction of general anesthesia, while no additional application was given to those in Group C. Patient-controlled intravenous analgesia (PCIA) with sufentanil was postoperatively performed in both groups. Sufentanil consumption and the Visual Analog Scale (VAS) score within 48 h postoperatively were assessed as primary outcomes. Secondary outcomes included peripheral levels of stress markers, intraoperative consumptions of anesthetic drugs and postoperative recovery.

**Results:**

Patients in Group B presented significantly lower VAS scores within 24 h and less consumption of sufentanil within 48 h postoperatively (both *p* < 0.01). The awaking time, time to extubation and length of stay were significantly shorter in Group B than in Group C (*p* = 0.005, 0.001 and 0.028, respectively). Compared with Group C, stress response and inflammatory response within 24 h postoperatively were also significantly milder in Group B.

**Conclusion:**

The use of buccal acupuncture before general anesthesia induction favors the postoperative analgesic effect and recovery in elderly patients undergoing laparoscopic radical gastrectomy, the mechanism of which involves relieving postoperative stress response and inflammatory response.

**Clinical trial registration:**

This study was registered in the Chinese Clinical Trial Registry (www.chictr.org.cn) on 15/06/2023 (ChiCTR2300072500).

## 1 Introduction

Laparoscopic radical gastrectomy is an effective minimally invasive procedure for the treatment of gastric cancer, but may cause moderate-to-severe abdominal wall pain and visceral pain that negatively affects postoperative rehabilitation (1). Appropriate postoperative pain management was beneficial for the patients’ recovery (2, 3). Patient-controlled intravenous analgesia (PCIA) with opioids was usually given to manage this postoperative pain (4). However, adverse events of PCIA like nausea, vomiting, lethargy, respiratory depression, skin pruritus and gastrointestinal dysfunction should be concerned (5). Kim et al. (6) showed that the combined use of non-steroidal anti-inflammatory drugs (NSAIDs) in patients treated with laparoscopic radical gastrectomy achieved an acceptable postoperative analgesia and a reduced consumption of postoperative opioids, but increased incidences of anastomotic leakage, duodenal stump leakage and intra-abdominal bleeding. Before anesthesia, rectus abdominis or rectus abdominis plane block combined with PCIA provides an effective postoperative analgesia for patients treated with laparoscopic radical gastrectomy (7, 8). However, nerve blocks have disadvantages of block failure, wrong-site blocks, nerve injury and local anesthetic poisoning. Postoperative epidural analgesia offers the same analgesic effect as PCIA on patients undergoing laparoscopic radical gastrectomy, whereas the former significantly increases the incidence of postoperative hypotension and urinary storage (9, 10). An effective and safe postoperative analgesia regimen is urgently needed for patients undergoing laparoscopic radical gastrectomy.

**Figure 1:**
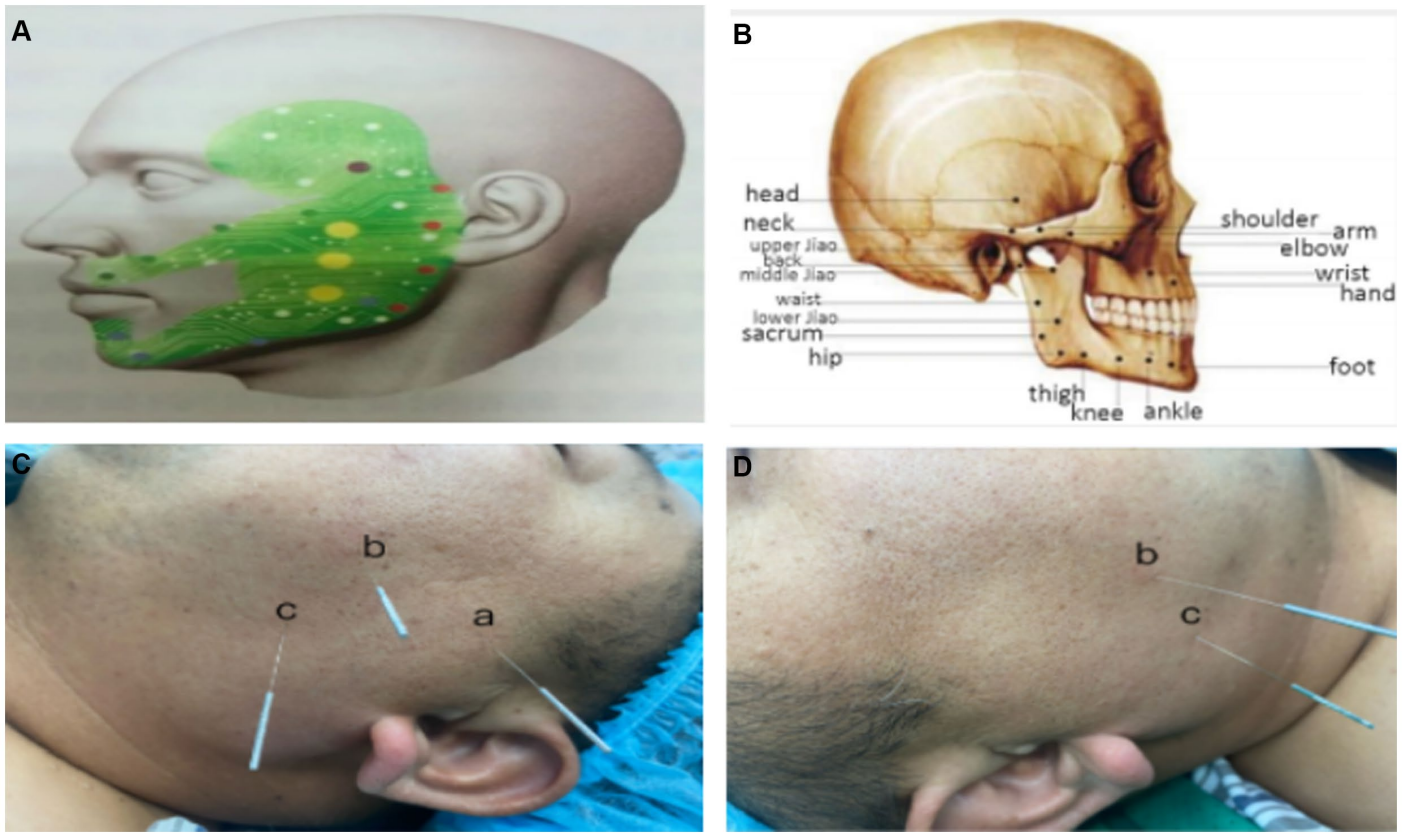
Buccal acupuncture and acupoints. **(A,B)** A biological holographic model of buccal acupuncture **(A)** (18), and acupoints on the cheek **(B)** (19). **(C,D)** Buccal acupoints of *a* (Shoulder point), *b* (Middle Jiao point), *c* (Lower Jiao point) on the left cheek **(C)**, and *b* (Middle Jiao point), *c* (Lower Jiao point) on the right cheek **(D)**. Acupoint *a* is situated on the midpoint of temporozygomatic suture for relieving the left shoulder pain; *b* is situated on the midpoint of Upper Jiao (the cross of the posterior coronoid of the mandible and the lower edge of the zygomatic arch) and Lower Jiao (anterior oblique line of the mandible); *c* is situated on the anterior oblique line of the mandible.

Postoperative pain, a type of inflammatory pain, is caused by peripheral tissue damage involving central and peripheral sensitization mechanisms (11, 12). Habibi et al. (13) and Ezzatvar et al. (14) indicated that central sensitization is the main cause of prolonged pain and poor analgesic effect. Perioperative acupuncture was reported to achieve the postoperative analgesic effect with less consumptions of analgesic drugs, by blocking harmful stimuli, reducing peripheral and central sensitization and inhibiting neuroplasticity (15). Acupuncture is a traditional Chinese medicine (TCM) technique with the recognized function in relieving pain (16, 17). The TCM theory believes that a congenital holographic system on the cheek is a continuous projection of the human body (Figure 1A) (18). Based on this, buccal acupuncture, as an emerging pain-free acupuncture therapy, stimulates 16 specific acupoints on the cheek to treat physical and mental diseases, including the chronic pain (Figure 1B). A growing body of evidence has supported the regulatory effects of buccal acupuncture on postoperative analgesia and viscera function with a high safety (19, 20). The present study explored the influence of buccal acupuncture before general anesthesia induction on postoperative analgesia and perioperative stress response in elderly patients undergoing laparoscopic radical gastrectomy and the underlying mechanism.

## 2 Methods

### 2.1 Study design

It was a prospective, outcome assessor-blinded, single-arm, randomized controlled trial (RCT) conducted by the Department of Anesthesiology in the Affiliated Jiangning Hospital of Nanjing Medical University, following the Consolidated Standards of Reporting Trials (CONSORT) statement (21). Ethical approval for this study (2023-03-019-K01) was provided by the Institutional Ethics Committee of the Affiliated Jiangning Hospital of Nanjing Medical University. This study was registered in the Chinese Clinical Trial Registry on 15/06/2023 (CTRI registration number—ChiCTR2300072500). Written informed consent was provided by all participants. Surgical procedures and follow-up within 48 h were performed by the same group of surgeons and a nurse anesthetist who were blinded to group allocation.

### 2.2 Participants

Ninety patients with an age of 65–80 years, body mass index (BMI) of 22–28 kg/m^2^, and the American society of Anesthesiologists (ASA) physical status of Class II – III who were treated with an elective laparoscopic radical gastrectomy were enrolled. Exclusion criteria: (1) cardiac, hepatic, pulmonary or renal insufficiency; (2) diabetes mellitus, neurological diseases, blood disorders or immune system disorders; (3) mental illnesses, hearing or language disorders; (4) medical history of chronic pain, history of psychotropic or analgesic medication, drug use or alcohol dependence; (5) acute head and facial injuries, soft tissue diseases, or infection; (6) suspected intubation difficulty with the Mallampati Scoring of Class II and above (22); (7) coagulation abnormalities or long-term use of anticoagulants; (8) intraoperative falling of the acupuncture needle; (9) intraoperative conversion to transabdominal surgery; (10) intraoperative bleeding greater than 500 mL or operative time longer than 5 h; (11) postoperative awaking time longer than 2 h or transferred to intensive care unit (ICU); (12) unusually sensitive to the pain or using tramadol for rescue analgesia more than 3 times.

### 2.3 Sample size

**Figure 2:**
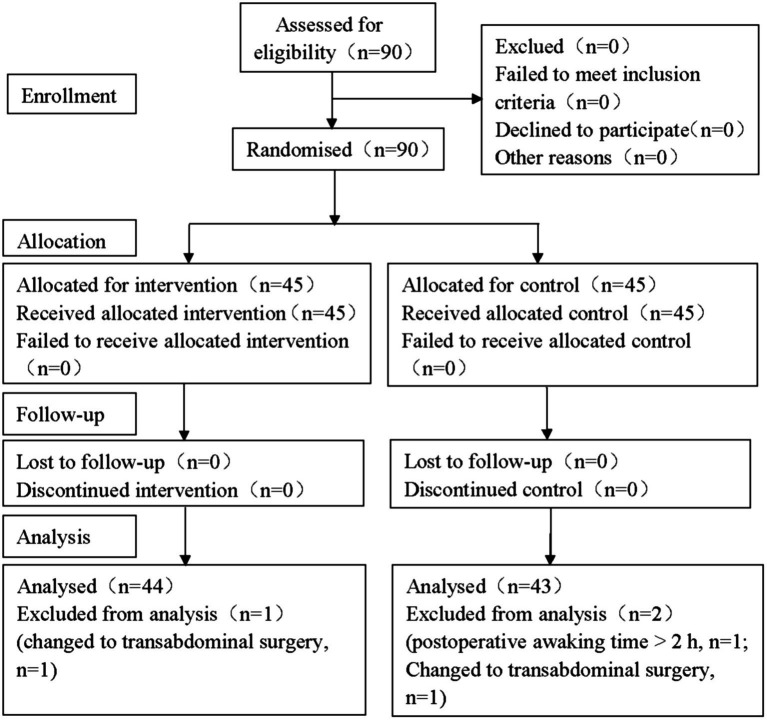
CONSORT flow diagram of participant enrollment. In the study, a total of 90 participants were initially screened and 3 of them were excluded during the trial. Finally, there were 87 participants included in the statistical analysis (43 in Group C and 44 in Group B, respectively). CONSORT: The Consolidated Standards of Reporting Trials.

Based on our pre-experiment with 10 participants per group, the mean consumption of sufentanil within 48 h postoperatively could be reduced to 9 μg (12%) in Group B. Assuming a 10% dropout rate at a power value of 80% and an *α* of 0.05, the sample size of at least 45 per group was needed. In the present study, 60 cases for each group were designed. A CONSORT flow diagram of participant enrollment was displayed in Figure 2.

### 2.4 Randomization and allocation concealment

Patients were randomly divided into two groups using a random table generated by SPSS 26.0. Ninety sealed envelopes were prepared by a statistician who did not participate in the study. To ensured that the assessor was not aware of the patients’ grouping, all patients’ head and face were covered with a disposable sterile medium and fixed using an anesthesia head frame. To assure concealment of allocation, the random number was kept in sealed and opaque envelopes, and opened up by an anesthesiologist who was not involved in this study.

### 2.5 Interventions

Patients in Group B were intervened by buccal acupuncture before general anesthesia induction by the same chief physician. After routine facial disinfection, five buccal acupuncture needles (Gauge 02, 0.18 mm × 30 mm, Maanshan Bond Medical Instruments Co., Ltd., China) were inserted to 5–15 mm depth for 30 min through five acupoints of the left shoulder point and bilateral points of Middle Jiao and Lower Jiao (Figures 1C, D) (23). No interventions were performed in Group C.

### 2.6 Anesthesia management

Preoperative fasting involved at least 12 h for food and 6 h for fluids. After entering the operating room, patients were managed by the peripheral intravenous placement and monitoring of electrocardiogram (ECG), heart beat (HR), pulse oxygen saturation (SpO_2_), end-tidal carbon dioxide partial pressure (P_ET_CO_2_) and bispectral index (BIS). Radial artery puncture and catheterization were performed under local anesthesia to monitor the invasive arterial blood pressure (IBP). After 5-min oxygen delivery at 8 L/min by an oxygen mask, general anesthesia induction was performed using midazolam (0.05 mg/kg), etomidate (0.15 mg/kg), cisatracurium (0.2 mg/mg) and sufentanil (0.4 μg/kg). Mechanical ventilation via tracheal cannula was implemented, with the mode of intermittent positive-pressure ventilation (IPPV), tidal volume (V_T_) of 8 mL/kg, respiratory rate (RR) of 14 times/min, ratio of the duration of inspiratory and expiratory phases (I:E ratio) of 1:2, inhaled oxygen concentration of 60% and intraoperative P_ET_CO_2_ of 35–45 mmHg. Anesthesia was maintained by intraoperative venous pumping of propofol (3–6 mg/kg/h), remifentanil (0.1–0.3 μg/kg/min) and cisatracurium (0.1–0.2 mg/kg/h), with BIS value of 45–60. Uradil (12.5 mg/time), neo-synephrine (100 μg/time), esmolol (30 mg/time) and atropine (0.25 mg/time) were given in cases of intraoperative hypertension (20% increase in the mean arterial blood pressure (MAP) or SBP > 160 mmHg), hypotension (20% decrease in MAP than baseline or SBP < 90 mmHg), tachycardia and bradycardia (HR <50 beats/min), respectively. The infusion of cisatracurium was terminated 30 min before surgery, and that of propofol and remifentanil was stopped at the last stitch of skin suture. The PCIA pump was connected with the intravenous line for 150 mL of normal saline containing sufentanil (2.0 μg/kg) and palosetron (0.25 mg), 5 mL of load volume, 2 mL/h of background dose and 1 mL of bolus dose. Tramadol (1 mg/kg) was additionally given to patients with a poor anesthetic effect of PCIA [postoperative Visual Analogue Scale (VAS) score > 3 points]. All patients were postoperatively transferred to postanesthesia care unit (PACU) for recovery. The tracheal catheter was extubated until the recovery of the consciousness, muscle strength, swallowing reflex and spontaneous breathing, V_T_ > 5 mL/kg and RR > 12 times/min. Patients with the Steward score of 4 points and above were transferred to the ward (24).

### 2.7 Outcome measures

The primary outcomes included sufentanil consumption within 48 h postoperatively and the VAS scores at 2 h (T_1_), 6 h (T_2_), 24 h (T_3_) and 48 h (T_4_) postoperatively.

The secondary outcomes included: (1) intraoperative consumptions of propofol, remifentanil and vasoactive drugs; (2) postoperative awakening time, time to extubation and length of stay; (3) number of presses of the PCIA device, number of effective presses and case number of rescue analgesia within 48 h postoperatively; (4) peripheral levels of blood glucose (BG), adrenaline (E), noradrenaline (NE), C-reactive protein (CRP), Cortisol (Cor), interleukin-6 (IL-6) and interleukin-10 (IL-10) before general anesthesia induction (T_0_) and at T_1_, T_2_, T_3_ and T_4_; (5) adverse events like postoperative nausea and vomiting (PONV), drowsiness, respiratory depression (SpO_2_ < 90%) and skin pruritus within 48 h postoperatively.

### 2.8 Statistical analysis

Data analysis was performed by the SPSS 26.0 (SPSS Inc., Chicago, IL, United States). Continuous variables were tested for normality, and those meeting the normal distribution were presented as mean ± standard deviation (SD). Differences between the two groups were analyzed by the independent samples *t*-test. Categorical variables were presented as numbers (%), and differences between groups were analyzed by the Chi-square or Fisher’s exact test. A statistically significant difference was considered with the *p* < 0.05.

## 3 Results

### 3.1 Enrollment of participants

A total of 90 participants were initially screened. After excluding 2 cases in Group C (one case of longer than 2 h of postoperative awaking, and one of intraoperative conversion to transabdominal surgery) and 1 case in Group B (intraoperative conversion to transabdominal surgery), 43 cases in Group C and 44 in Group B were finally enrolled in our study (Figure 2).

### 3.2 Baseline characteristics

There were no significant differences in baseline characteristics between Group B and Group C, including sex, age, body mass index (BMI), ASA classification, operative time, intraoperative bleeding and intraoperative infusion volume (all *p* > 0.05, Table 1).

**Table 1:** Baseline characteristics of participants (*n* = 87).

Characteristic	Group C (*n* = 43)	Group B (*n* = 44)	*p*-value
*^a^Gender (n, %)*			
Female	17 (39.5)	20 (45.5)	0.173
Male	26 (60.5)	24 (54.5)	0.228
^b^Age (years), mean ± SD	71.3 ± 5.6	73.6 ± 6.7	0.371
^b^BMI (kg/m^2^), mean ± SD	26.4 ± 3.5	24.9 ± 4.8	0.248
*^a^ASA physical status (n, %)*			
Class II	18 (41.9)	16 (36.4)	0.749
Class III	25 (58.1)	28 (63.6)	0.322
^b^Operative time (min), mean ± SD	206.2 ± 18.9	198.7 ± 21.2	0.516
^b^Intraoperative bleeding (ml), mean ± SD	98.5 ± 6.2	101.3 ± 7.4	0.293
^b^Intraoperative infusion (ml), mean ± SD	2250.8 ± 98.6	2135.2 ± 105.3	0.334

SD, standard deviation; BMI, body mass index; ASA, American Society of Anesthesiologists. ^a^Comparison based on *χ*^2^ test. ^b^Comparison based on an independent sample *t*-tests.

### 3.3 Intraoperative consumptions of anesthetic drugs

Compared with those of Group C, intraoperative consumptions of propofol, remifentanil, neo-synephrine, urapidil, esmolol and atropine were significantly lower in Group B (*p* < 0.05, Table 2).

**Table 2:** Intraoperative consumptions of analgesic drugs (mean ± SD).

	Group C (*n* = 43)	Group B (*n* = 44)	*p*-value^a^
Propofol (mg)	926.8 ± 22.3	798.1 ± 19.4	0.003
Remifentanil (μg)	967.7 ± 31.5	783.4 ± 27.6	0.006
Neo-synephrine (μg)	41.9 ± 4.2	22.7 ± 3.8	0.005
Urapidil (mg)	4.4 ± 0.8	1.7 ± 0.5	0.001
Esmolol (mg)	8.4 ± 2.6	4.1 ± 1.7	0.006
Atropine (μg)	58.1 ± 7.9	34.1 ± 5.2	0.033

SD, standard deviation. ^a^Comparison based on an independent sample *t*-tests.

### 3.4 Postoperative pain

Compared with those of Group C, patients in Group B were graded with significantly lower VAS scores at T_1_, T_2_ and T_3_ (*p* < 0.05), but shared similar VAS scores at T_4_ (*p* > 0.05, Table 3).

**Table 3:** Postoperative VAS scores (mean ± SD, score).

	Group C (*n* = 43)	Group B (*n* = 44)	*p*-value^a^
T_1_ (points)	1.6 ± 0.6	0.9 ± 0.3	0.011
T_2_ (points)	1.8 ± 0.4	1.0 ± 0.4	0.018
T_3_ (points)	1.9 ± 0.7	1.2 ± 0.5	0.023
T_4_ (points)	2.1 ± 0.6	1.9 ± 0.5	0.656

SD, standard deviation; VAS, Visual Analogue Scale; T_1_, 2 h postoperatively; T_2_, 6 h postoperatively; T_3_, 24 h postoperatively; T_4_, 48 h postoperatively. ^a^Comparison based on an independent sample *t*-tests; *p*-value significant (i.e., <0.05) indicated in bold.

### 3.5 Secondary outcomes

The number of presses of the PCIA device, number of effective presses and case number of rescue analgesia within 48 h postoperatively were significantly lower in Group B than in Group C (*p* < 0.05, Table 4). Moreover, sufentanil was significantly less consumed in Group B (*p* < 0.01, Table 4).

**Table 4:** Postoperative analgesic indices.

	Group C (*n* = 43)	Group B (*n* = 44)	*p*-value
^a^PCIA press (*n*), mean ± SD	8.1 ± 2.0	4.5 ± 1.4	0.006
^a^Effective press (*n*), mean ± SD	7.6 ± 1.8	3.9 ± 1.2	0.009
^b^Rescue analgesia (*n*, %)	7 (16.3)	2 (4.5)	0.002
^a^Sufentanil consumption (μg), mean ± SD	77.6 ± 6.8	68.2 ± 5.3	0.023

PCIA, patient-controlled intravenous analgesia; SD, standard deviation. ^a^Comparison based on an independent sample *t*-tests; ^b^Comparison based on *χ*^2^ test; *p*-value significant (i.e., <0.05) indicated in bold.

At T_1_, T_2_ and T_3_, significantly lower peripheral levels of BG, E, NE, CRP, Cor and IL-6, and higher IL-10 level were detected in Group B compared with those of Group C (*p* < 0.01, Table 5). However, no significant differences in them were detected at T_0_ and T_4_ between groups (*p* > 0.05).

**Table 5:** Postoperative blood testing in Group C (*n* = 43) and Group B (*n* = 44) postoperatively (mean ± SD).

	Group	*T*_0_	*T*_1_	*T*_2_	*T*_3_	*T*_4_
BG (mmol/L)	Group C	5.3 ± 1.4	7.6 ± 2.3	8.1 ± 2.5	6.7 ± 2.4	5.6 ± 1.7
	Group B	5.6 ± 1.8	6.5 ± 2.1	6.8 ± 2.3	5.7 ± 1.6	5.5 ± 2.2
	*p*-value^a^	0.618	0.008	0.003	0.005	0.327
E (pmol/L)	Group C	326.2 ± 18.3	538.4 ± 23.7	585.4 ± 26.2	532.7 ± 22.8	486.5 ± 19.1
	Group B	331.7 ± 16.5	486.5 ± 19.6	519.8 ± 21.5	480.8 ± 17.4	469.6 ± 18.7
	*p*-value^a^	0.870	0.006	0.001	0.002	0.656
NE (nmol/L)	Group C	3.5 ± 1.2	4.6 ± 1.8	5.1 ± 1.9	4.5 ± 1.6	3.7 ± 1.4
	Group B	3.3 ± 1.1	3.8 ± 1.3	4.2 ± 1.4	3.6 ± 1.3	3.4 ± 1.3
	*p*-value^a^	0.226	0.005	0.006	0.003	0.429
CRP (mg/L)	Group C	12.7 ± 3.4	23.8 ± 5.7	28.2 ± 6.9	33.6 ± 6.5	25.4 ± 4.4
	Group B	13.3 ± 3.9	17.2 ± 4.1	21.4 ± 5.3	26.3 ± 6.0	23.2 ± 3.7
	*p*-value^a^	0.928	0.001	0.007	0.002	0.858
Cor (ng/ml)	Group C	68.2 ± 7.7	79.6 ± 8.4	85.5 ± 9.1	93.4 ± 11.2	81.5 ± 8.2
	Group B	66.7 ± 8.1	71.4 ± 7.9	77.2 ± 7.8	83.1 ± 9.6	78.3 ± 8.9
	*p*-value^a^	0.870	0.006	0.001	0.002	0.656
IL-6 (pg/ml)	Group C	23.6 ± 4.7	71.3 ± 8.6	63.5 ± 7.4	54.8 ± 5.9	39.2 ± 4.1
	Group B	25.5 ± 5.1	60.2 ± 6.7	53.1 ± 5.3	42.7 ± 4.6	37.5 ± 4.3
	*p*-value^a^	0.393	0.005	0.003	0.006	0.558
IL-10 (pg/ml)	Group C	8.1 ± 2.6	11.5 ± 3.2	23.6 ± 4.8	30.3 ± 6.1	26.8 ± 5.5
	Group B	7.8 ± 3.1	18.2 ± 4.7	31.5 ± 6.2	38.7 ± 6.4	28.3 ± 4.9
	*p*-value^a^	0.296	0.002	0.005	0.001	0.612

SD, standard deviation; BG, blood glucose; E, adrenaline; NE, noradrenaline; CRP, C-reactive protein; Cor, cortisol; IL-6, interleukin 6; IL-10, interleukin 10. T_1_, 2 h postoperatively; T_2_, 6 h postoperatively; T_3_, 24 h postoperatively; T_4_, 48 h postoperatively. ^a^Comparison based on an independent sample *t*-tests; *p*-value significant (i.e., <0.05) indicated in bold.

Significantly shorter postoperative awakening time, time to extubation and length of stay in Group B than those of Group C were detected (*p* < 0.05, Table 6). The incidence of PONV within 48 h postoperatively was significantly lower in Group B than in Group C (*p* < 0.01), while those of drowsiness, respiratory depression and skin pruritus were similar (*p* > 0.05; Table 6).

**Table 6:** Postoperative recovery and adverse events.

	Group C (*n* = 43)	Group B (*n* = 44)	*p*-value
*^a^Postoperative recovery (mean ± SD)*			
Awakening time (min)	26.7 ± 5.5	19.8 ± 4.2	0.005
Time to extubation (min)	28.3 ± 6.1	21.5 ± 4.9	0.001
Length of stay (days)	9.1 ± 1.8	7.2 ± 1.5	0.028
*^b^Adverse events (n, %)*			
PONV	12 (27.9)	5 (11.4)	0.003
Drowsiness	2 (4.7)	3 (6.8)	0.898
Respiratory depression	1 (2.3)	0	0.969
Skin pruritus	1 (2.3)	2 (4.5)	0.627

SD, standard deviation; PONV, post-operative nausea and vomiting. ^a^Comparison based on an independent sample *t*-tests; ^b^Comparison based on Fisher’s exact test; *p*-value significant (i.e., <0.05) indicated in bold.

## 4 Discussion

Buccal acupuncture is a new type of microneedling created by Wang Yongzhou (23), abiding by the theory that the physiology of the human body can be projected onto a holographic acupoint system visualized on the cheek. In a clinical trial involving 630 patients with limb pain and 100 patients with visceral pain, buccal acupuncture was validated for its rapid, reliable and systemic therapeutic efficacy. Moreover, various races can benefit from buccal acupuncture (18). In the present study, we proved that the use of buccal acupuncture before general anesthesia induction significantly reduced the consumption of intraoperative anesthetic drugs and postoperative analgesic drugs, and alleviated perioperative stress response and inflammatory response in elderly patients undergoing laparoscopic radical gastrectomy.

The 6th–10th thoracic vertebrae (T_6_–T_10_) provide the sensory innervation of the stomach, causing abdominal wall and visceral pain after surgery (25). Appropriate postoperative analgesia methods were conducive to patients’ recovery (2, 3). The traditional Chinese medicine (TCM) theory believed that the liver, gallbladder, spleen and stomach are harbored in the Middle Jiao, and the lower abdomen and urogenital reproduction in the Lower Jiao. Yi et al. (20) showed that the aging curve of the analgesic effect of buccal acupuncture was inherent and specific, and did not change with the number of treatments. In addition, they reported an obvious analgesic effect of buccal acupuncture starting at 5 min and peaking at 30 min. The analgesic effect of buccal acupuncture manifested as a temporal change with the rapid onset of analgesia, and increased in pain threshold and stability without being influenced by multiple times of acupunctures. We performed buccal acupuncture on the acupoints of the left shoulder point, bilateral acupoints of Middle Jiao and Lower Jiao for 30 min before general anesthesia induction in patients treated with laparoscopic radical gastrectomy. Consistent with previous data, the VAS scores within 24 h postoperatively were significantly lower in patients treated with buccal acupuncture than those of controls (17).

Surgical procedures of laparoscopic radical gastrectomy, anesthesia stimulation, establishment of CO_2_ pneumoperitoneum and hypercapnia resulted in perioperative stress response, intraoperative hemodynamic fluctuations and postoperative pain (11, 12). Kurni et al. (26) verified that stress-related indicators like plasma Cor level are positively correlated with the intensity and duration of stimuli. The use of buccal acupuncture before general anesthesia induction significantly lowered peripheral levels of BP, E, NE, Cor and CRP within 24 h postoperatively, indicating the relieved postoperative stress response. Surgical trauma aggravated postoperative pain and inflammatory response by releasing inflammatory mediators, which could be alleviated by an effective preoperative regional anesthesia to inhibit the transmission of trauma and pain stimulation to the advanced center (14, 27). IL-6 is the earliest released and highly sensitive proinflammatory factor, closely related to the severity of surgical trauma, body immune status and prognosis (28). IL-10, as an inflammatory suppressor, inhibits nuclear factor activity and promotes neutrophil apoptosis (29). In this study, decreased IL-6 and increased IL-10 levels within 24 h postoperative in Group B suggested the role of buccal acupuncture in balancing the pro- and anti-inflammatory signals.

The regulatory effect of buccal acupuncture on inflammatory response is probably attributed to the blockage of pain signal to the central nervous system (15). Wang et al. (17) demonstrated the synergistic analgesic effect of acupuncture by relieving oxidative stress and inflammatory response, with the direct or indirect involvement of the nervous and humoral system. In a white rabbit model of rheumatoid arthritis (30), buccal acupuncture effectively relieved the pain by affecting the release of β-endorphin (β-EP) and cholecystokinin octapeptide (CCK-8) in cerebrospinal fluid. β-EP produces the analgesic effect by blocking pain signals via sensitizing the endogenous opioid peptide (EOP) receptors in neuronal terminals, or activating receptors with gray matter distribution around the brainstem aqueduct (31, 32). CCK-8 is a strong endogenous anti-opioid peptide with the central anti-opioid analgesic effect. It contributes to pain relief through reducing the total number of μ receptors, decreasing the affinity of κ receptors and inhibiting the binding force between opioid and receptors (33). Characterized by the analgesic time-effect, the use of buccal acupuncture in rabbits increased the content of 5-HT and decreases the ratio of NE/5-HT in the hypothalamus (30, 34).

The safety profile of buccal acupuncture was examined. There were no significant differences in the incidences of drowsiness, respiratory depression and skin pruritus between groups. The significantly lower incidence of PONV in Group B than in Group C could be explained by the greater consumption of postoperative analgesic drugs when PCIA was used alone (35). Additionally, blockage of Qi movement in the Middle Jiao caused gastrointestinal symptoms (36). We believed that buccal acupuncture on bilateral acupoints of the Middle Jiao effectively removed Qi stagnation and thus relieved PONV (37).

There were some limitations in this study. First of all, it was a prospective, outcome assessor-blinded RCT. We failed to find a suitable placebo in the control group to achieve double blinding. However, during the operation, all patients’ head and face were covered by a surgical scarf and head frame, so that the assessor was not aware of the patients’ grouping. Secondly, the exclusion of patients with suspected intubation difficulty (Mallampati Scoring of Class II and above) may cause potential biases. At last, the use of tramadol for postoperative salvage analgesia may influence the occurrence of PONV. Taken together, buccal acupuncture reduces perioperative consumption of analgesic drugs and favors the postoperative analgesic effect and recovery in elderly patients undergoing laparoscopic radical gastrectomy with a high safety. This method can provide reference for postoperative analgesia in other surgical patients.

## 5 Conclusion

In conclusion, the use of buccal acupuncture before general anesthesia induction reduces perioperative consumption of analgesic drugs and favors the postoperative analgesic effect and recovery in elderly patients undergoing laparoscopic radical gastrectomy by relieving postoperative stress response and inflammatory response, which acceptable efficacy and safety.

## Data availability statement

The raw data supporting the conclusions of this article will be made available by the authors, without undue reservation.

## Ethics statement

The studies involving humans were approved by Medical Ethics Committee of Jiangning Hospital affiliated to Nanjing Medical University. The studies were conducted in accordance with the local legislation and institutional requirements. The participants provided their written informed consent to participate in this study.

## Author contributions

D-xZ: Data curation, Methodology, Visualization, Investigation, Writing – original draft. Y-lY: Investigation, Conceptualization, Methodology, Writing – original draft. LY: Data curation, Investigation, Writing – original draft. Y-yZ: Investigation, Methodology, Writing – original draft. Y-yX: Data curation, Software, Writing – original draft. WW: Conceptualization, Funding acquisition, Methodology, Project administration, Writing – review & editing. JL: Conceptualization, Formal analysis, Supervision, Writing – review & editing. W-yY: Supervision, Validation, Writing – review & editing.
